# Antimicrobial Use in the Animal Sector in Japan from 2011 to 2022

**DOI:** 10.3390/antibiotics13121204

**Published:** 2024-12-10

**Authors:** Yuta Hosoi, Mari Matsuda, Michiko Kawanishi, Saki Harada, Mio Kumakawa, Hideto Sekiguchi, Tetsuo Asai, Tatsuro Sekiya

**Affiliations:** 1 Veterinary AMR Center, National Veterinary Assay Laboratory, Ministry of Agriculture, Forestry and Fisheries, Tokyo 185-8511, Japan; 2 The United Graduate School of Veterinary Sciences, Gifu University, 1-1 Yanagito, Gifu 501-1193, Japan; 3 Assay Division II, National Veterinary Assay Laboratory, Ministry of Agriculture, Forestry and Fisheries, Tokyo 185-8511, Japan

**Keywords:** AMU, AMR, biomass

## Abstract

**Background/Objectives:** Evaluating antimicrobial use (AMU) is essential in the investigation and implementation of antimicrobial resistance (AMR) prevention measures. Here, we examined AMU using an index (mg/kg biomass) that considers the antimicrobial sales volume and livestock biomass in Japan from 2011 to 2022. **Methods:** Antimicrobial sales volumes were obtained from JVARM data, and biomass data were obtained from reliable statistics. Beef cattle, dairy cattle, pigs, broiler chickens, layer chickens, fish raised in freshwater, and fish raised in seawater were targeted in this study. **Results:** Tetracycline accounted for 39%, macrolides for 18%, penicillins for 12%, and sulfonamides for 11% of the sales in 2022. The peak antimicrobial sales volume was 847 tons in 2017, and then declined to 766 tons by 2022 with fluctuations in the interim. From the perspective of mg/kg biomass, AMU tended to increase in beef cattle, dairy cattle, and fish raised in seawater, while pigs, broilers, layers, and fish raised in freshwater showed a decreasing trend. In broilers, the decreasing trend that could not be confirmed by sales amount alone was detected using the newly established index. **Conclusions:** By calculating the mg/kg biomass, it became possible to create an interpretation that is different from that of the simple sales quantity data. We believe that this indicator is stable, transparent, and easily understandable for national monitoring.

## 1. Introduction

Veterinary antimicrobial products are essential for protecting animal health, ensuring stable supply lines of livestock and aquatic products to consumers, and treating animal infectious diseases; however, there is always a risk to humans, livestock, pets, and aquatic animals due to the emergence of antimicrobial-resistant bacteria caused by selective pressure from antimicrobial overuse [1,2]. The issue of antimicrobial-resistant bacteria has become one of the most important issues internationally, leading to the World Health Organization (WHO) formulating the Global Action Plan on antimicrobial resistance in 2015. This plan requires member countries to have national action plans on antimicrobial resistance that are aligned with the Global Action Plan [3]. In Japan, the National Action Plan on Antimicrobial Resistance was formulated in 2016 [4], and a number of measures were implemented based on it. Currently, the second period “2023–2027” of the National Action Plan is in progress, and the relevant experts, ministries, agencies, etc. are working together to promote measures against antimicrobial resistance. In this period of the action plan, one of the major outcome indicators set is a reduction in the use of antibacterial agents in the livestock industry by 15% from 2020 to 2027 [5]. Internationally, as part of its measures against AMR in the animal sector, the World Organisation for Animal Health (WOAH) collects, compiles, and publishes reports on the use of antimicrobial agents from various countries through their ANIMUSE system [6]. The Japanese Ministry of Agriculture, Forestry and Fisheries has been implementing the Japanese Veterinary Antimicrobial Resistance Monitoring System (JVARM) to monitor antimicrobial resistance (AMR) and antimicrobial use (AMU), publishing the findings annually. Risk control measures have been formulated and implemented based on the findings of JVARM, consisting of three pillars of analysis: antimicrobial use, the resistance status of bacteria from healthy animals, and the resistance status of bacteria from diseased animals [7]. Monitoring AMU in animals, in conjunction with AMR surveillance, provides essential information to empower appropriate veterinary services, leading to a reduction in the risk related to AMR in animal health and beyond [8]. Major indicators of AMU in the animal field include the weight of antimicrobial agents sold [6], methods that take dose into account (Defined Daily Dose (DDD) and Defined Course Dose (DCD)) [9], and methods that take animal weights into account as biomass (Population Correction Unit (PCU) [10], target animal biomass (TAB) [11], mg/kg biomass [6], etc.). In Japan’s AMR One Health report, the weight of the active ingredient in veterinary antimicrobials sold is used as an indicator of AMU [12]. PCU, which estimates the body weight of animals at the time of treatment to calculate the biomass, is used in several countries and institutions, including the EU [10], Canada [13], and Thailand [14]. This takes into account the various weights of animals in that category at the age that they would most likely be treated with antimicrobials [15]. The DDD for which the daily dose of antimicrobial agents can be considered is used in Denmark [16] and the Netherlands [17], and the DCD for which the dose used for a single treatment can be considered is used partially in France under the name ALEA [18]. A common disadvantage of each of these methods is that many assumptions need to be made to calculate each indicator. For example, for PCU, dairy cattle of an average weight at the time of treatment are estimated to be 425 kg as in ESVAC [10], but the assumption of this weight is not valid in other countries because practices of farming differ between countries. Though some countries still use PCU, weight assumptions were tailored to their circumstances (Canadian PCU [13], PCU Thailand [14]). In addition, considering that the mean Japanese dairy cattle weight was 639 kg in 2022 [19], it is assumed that they differ greatly from the ESVAC assumptions. By changing the assumption of body weight, the results of the calculations differ greatly. On the other hand, the WOAH uses mg/kg biomass to enable comparability between member states [6]. The methodology was devised to create a means of uniform comparison between countries, using globally available statistical data (such as the FAO Statistical Database) to standardize the calculations. The WOAH method and the method employed in the present study differ in terms of the biomass calculation method. The methodology used in this study works to eliminate assumptions as much as possible when estimating the biomass, utilizing documented national core statistics. The WOAH method for calculating mg/kg biomass has advantages for cross-country comparisons, but we believe that the method in this study has advantages for monitoring long-term trends within one country, Japan. Cross-country comparisons using AMU help in understanding their objective rankings, but it is more important for each country to consistently track its AMU over time using stable methods. To that end, we analyzed data for the country of Japan by tallying antimicrobial sales data and calculating mg/kg biomass based on the data published in Japan, separated by the class of antimicrobials and animal species. The way we approach this, through analyzing mg/kg biomass, allows us to normalize the antimicrobial sales volumes by animal weight and then take that further to analyze the degree of exposure of antimicrobials to animals, which cannot be determined solely by the antimicrobial sales volume. In other words, in terms of sales volume alone, the number of livestock fluctuates year to year due to numerous situations surrounding livestock populations, such as animal husbandry, the occurrence of diseases, etc.; however, the index created here is not influenced by such fluctuations. This enables us to obtain a more accurate interpretation of AMU per animal species and insight on the effects of preventive measures against AMR in each animal species. In this study, the species targeted were the most prevalent livestock in Japan: beef cattle, dairy cattle, pigs, layers, broilers, fish raised in freshwater, and fish raised in seawater.

## 2. Results

### 2.1. Sales Amounts of Antimicrobial Agents

**Figure 1:**
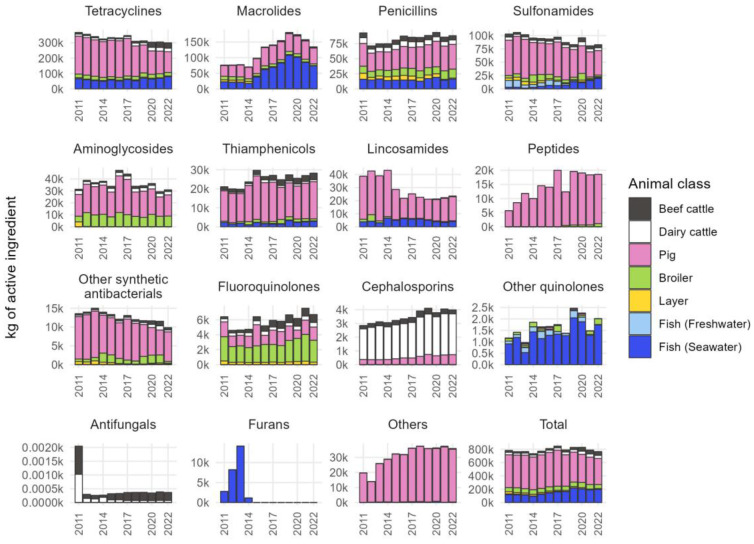
Trends of quantities of veterinary antimicrobial agents sold for therapeutic use in Japan by antimicrobial class.

The sales amounts of veterinary antimicrobial agents sold for use in livestock in Japan from 2011 to 2022, calculated to only account for the active ingredients’ weights, are shown by antimicrobial class in Figure 1.

From 2011 to 2022, the smallest sales amount was 734 tons in 2014 while the largest was 847 tons in 2017. After the peak in 2017, the amount has fluctuated but ultimately decreased to 766 tons by 2022.

Among them, tetracyclines, macrolides, penicillins, and sulfonamides accounted for the majority. In 2022, tetracyclines accounted for 39%, macrolides 18%, penicillins 12%, and sulfonamides 11% of the total sales (Supplemental Table S1). Although tetracyclines are the most used antimicrobial agents in livestock, the sales of tetracyclines have gradually decreased since 2011. Macrolides increased from 2015 and had a peak sales volume in 2019, but since then, this has declined through 2022. Penicillins have gradually increased in sales volume from 2012 to 2022. Sulfonamides have been progressively decreasing in sales since 2011. Although the sales volumes of cephalosporins and fluoroquinolones, which are important antimicrobial agents for human medicine, remained low, they have been generally increasing. The estimated marketed proportion of animals varies among the antimicrobial classes; however, it can be seen that the proportions of antimicrobials used in pigs for many classes of antimicrobials are high. The propensity differs from that of macrolides, other quinolones, which have higher rates of use in fish raised in seawater, aminoglycosides and fluoroquinolones, which have higher proportions of broilers, and cephalosporins in dairy cattle.

Animal-specific sales volumes of veterinary antimicrobial agents converted to active ingredient weights are shown in Table 1. Sales for pigs, fish raised in seawater, and broilers had the greater proportions, accounting for 51%, 26%, and 8% of the total sales in 2022, respectively. Antimicrobial sales for pigs peaked in 2017 and declined by 150 tons between 2017 and 2022. Table 1 also shows the proportion of the weight of antimicrobials administered orally. The proportion of oral administration in beef cattle was approximately 80%, in dairy cattle 70%, and in other animals, it was over 95%.

### 2.2. Biomass

Table 2 shows the estimated biomass from 2011 to 2022. The estimated total biomass in Japan increased from 5.061 M tons in 2011 to 5.469 M tons in 2022. In 2022, the biomass was 2.224 M tons in broilers, 1.293 M tons in pigs, 0.876 M tons in dairy cattle, 0.491 M tons in beef cattle, 0.316 M tons in layers, 0.237 M tons in fish raised in seawater, and 0.031 M tons in fish raised in freshwater.

### 2.3. AMU Index (mg/kg Biomass)

**Figure 2:**
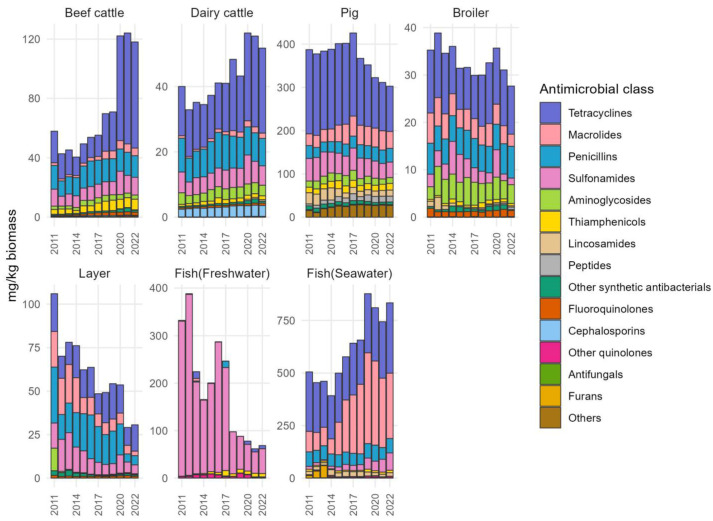
Trends of quantities of veterinary antimicrobial agents sold for therapeutic use in Japan in mg/kg biomass of the respective target species.

The estimated sales of antimicrobials were divided by biomass per animal from 2011 to 2022 are shown in Figure 2 (detailed tables are in Supplemental Table S2). There was an increasing trend in mg/kg biomass in beef cattle, dairy cattle, and fish raised in seawater, and a decreasing trend in mg/kg biomass in broilers, layers, and fish raised in freshwater. In pigs, a decreasing trend was observed starting from 2017. As for beef cattle, the mg/kg biomass of tetracyclines increased from 2019 to 2020. With regard to pigs, there was a marked downward trend from 2017 onwards. Especially, the ratio of tetracyclines decreased remarkably. Though the ratio of penicillin was high in layers, it has recently taken on a decreasing trend. In fish raised in freshwater, sulfonamides are often used, but that amount dropped noticeably for a moment from 2017 to 2018. Regarding fish raised in seawater, the period between 2014 and 2019 saw a rise in macrolide use, which resulted in an elevated total antimicrobial consumption that remained high until 2022.

### 2.4. Trend Analysis of mg/kg Biomass

**Figure 3:**
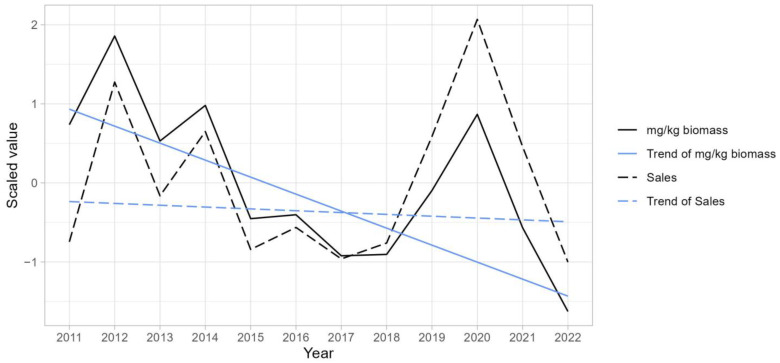
Trends of quantities of veterinary antimicrobial agents sold and mg/kg biomasses of broilers. The values are normalized. The trend lines are constructed using Sen’s slope and are designed to pass through the point (x = 2016.5, y = median of the scaled values).

There are differences between the trends in the sales amount and mg/kg biomass. The trends of antimicrobial sales and mg/kg biomasses of broilers are standardized to enable a comparison regardless of the units and shown in Figure 3. While sales trends appear to repeatedly fluctuate, there appears to be a decreasing trend in mg/kg biomass. In order to confirm the existence of the trend statistically, the Mann–Kendall trend test was conducted. When we set the significance level at 0.05, it was judged that there was no trend with a *p*-value of 0.63 on the sales quantity; conversely, with a *p*-value of 0.047 on mg/kg biomass, it was judged that there was a notable trend. Subsequently, Sen’s slope was used to calculate the slope of the trend for the standardized values. The general slope of the trend for the sales was −0.02, while mg/kg biomass was −0.22, with a larger decreasing trend seen in mg/kg biomass. Similarly, in some of the antimicrobial class and animal combinations, there were instances where discrepancies arose in the assessment of trends between the sales volume and mg/kg biomass. For example, cephalosporins in beef cattle expressed a Mann–Kendall *p*-value of 0.09 in sales (Sen’s slope; 0.23) that is judged as not having a trend, but mg/kg biomass yielded a *p*-value of 0.047 (Sen’s slope; 0.23), indicating that there was a slight upward trend. Fluoroquinolones in broilers had a *p*-value of 0.03 for the sales volume (Sen’s slope; 0.17) and a *p*-value of 0.3 for mg/kg biomass (Sen’s slope; 0.09), while penicillins had a *p*-value of 0.005 for the sales volume (Sen’s slope; 0.24) and a *p*-value of 0.15 for mg/kg biomass (Sen’s slope; 0.11) in the trend test. Thus, fluoroquinolones and penicillins were judged to be on an upward trend based on the sales amount, but not on a mg/kg biomass basis.

## 3. Discussion

The latest national action plan on antimicrobial resistance, formulated in 2023, sets a new target for reducing the use of antimicrobial agents in the livestock sector. By 2027, the goal is to decrease antimicrobial agent use by 15% compared with the levels observed in 2020. As of 2022, these levels have already decreased by 9%, but it is necessary to continue to reduce the use of antimicrobial agents [5]. We attempted to forecast sales volumes for 2027 and estimate the rate of decline from 2020, using sales data from 2020 to 2022 without considering other variables; however, the results varied widely depending on the model used. An automatic ARIMA model, an exponential smoothing state space model, and a linear model predicted, respectively, a 4%, 21%, and 27% reduction, with their respective 95% prediction intervals being [11.8%, −3.4%], [50.7%, −7.3%], and [74.9%, −20.3%]. All of these have large prediction intervals and thus it is difficult to predict accurately.

Regarding beef cattle, the amount of tetracyclines sold increased from 2019 to 2020, but the detailed reasons are unclear since JVARM reports on the amount sold are based on tabulations of reports from marketing authorization holders. In dairy cattle, the ratio of cephalosporins is higher than in other animals because cephalosporins are used frequently in the form of udder injections to treat mastitis. Future measures, such as the prevention of mastitis by non-antibiotic measures (i.e., improvement in the sanitary conditions for livestock farming and milking hygiene, vaccinations, and teat sealant), are expected to reduce the amount used. In pigs, the reduction in AMU from 2017 to 2022 may be due to successful controls implemented by farmers and veterinarians combined with the success of awareness activities promoting prudent use based on the national action plan. In addition, swine fever, which had not occurred since 1992, had several confirmed cases in September 2018, leading to farmers performing more rigorous biosecurity and hygiene management. This may have resulted in better livestock hygiene and fewer situations in which antimicrobials must be used. However, reduction in AMU in pigs continues to be one of the prioritized components in the measures against AMR in livestock, and sustained commitment to prevent disease intrusion is important. Antimicrobial agent sales for fish raised in seawater have been increasing since 2015 and peaked in 2019. This is mainly due to increased macrolides sales volume caused by an outbreak of an infectious disease from type II alpha hemolytic streptococcosis which differed from the conventional serotype in which the vaccine was effective. The improvements in vaccines and additional strategies may have led to a change in the trend from rising to falling in 2020.

In JVARM, the percentage of antimicrobial-resistant bacteria of animal origin is monitored in addition to the amount of antimicrobial substances sold. With the exception of a few outlying years, resistance rates of tetracyclines tend to be the highest among the antimicrobial agents in *E. coli* from healthy cattle, pigs, and chickens [7]. This may be due to tetracyclines making up the greatest amount of AMU currently on the market. In swine, the sales volume of tetracyclines has been decreasing, but the resistance rate has not been reduced [20]. This deviates from the AMR monitoring carried in the Netherlands, which noted that decreases in the AMR in isolates from most livestock species happened simultaneously after decreases in the veterinary antimicrobial use, with the exception of tetracycline and fluoroquinolone resistance in campylobacter in poultry [17]. The differences observed between the Netherlands’ data and Japan’s data can be attributed to several factors. It has been suggested that the administration of other drugs may induce cross-resistance and co-resistance, which is associated with tetracycline resistance, potentially explaining why the rate of tetracycline resistance has not decreased [21]. Additionally, although the use of chloramphenicol in food-producing animals was banned in Japan in 1998, chloramphenicol-resistant strains were found in cattle and pig samples from 2001 to 2004, indicating that the possibility of co-resistance and resistance persisted despite the ban [22]. In addition, it has been indicated that the relationship between the tetracycline AMU and AMR can be modeled as an upward convex quadratic function with AMU as the variable [23]. The current level of AMU in pigs might be still at a point where the slope of the tangent to the quadratic function is relatively gentle, which may explain the lack of an observed reduction in AMR. In 2022, the sales volume of fluoroquinolones was highest for broilers, accounting for 44%. This corresponds to the observed resistance rate of fluoroquinolones in broilers being higher than in other livestock, reaching 22% in 2020 [20]. In the national action plan, the target for the resistance rate of chicken fluoroquinolones was set at 15% or less in 2027 [5], and fluoroquinolones should be used with greater caution as second-line drugs.

The AMU unit used in this study, mg/kg biomass, is a preferable indicator as a national monitoring index because it is an intuitively easy-to-understand index that avoids making assumptions whenever possible. We speculate that simplicity and comprehensibility have not been well evaluated as an indicator of AMU so far, but we believe that these are crucial elements that are needed to create public understanding (including in farmers and veterinarians). Our method also has the advantage of being able to eliminate assumptions as much as possible and obtain stable values. However, other indicators might be more suitable for benchmarking the comparisons between farms because differences in doses in drugs cannot be considered using this method. Abe et al. are actively evaluating AMU based on the DDD in Japan. They observed that, since tetracyclines and sulfonamides are administered at higher doses, the percentages of these antimicrobials calculated by the DDD index are relatively low among antimicrobials compared with the sales weight index. They also highlight the necessity to use the Japanese defined daily doses because the dosages used in Japan are different from those of the European Medicines Agency [24,25]. Another limitation of this study is that, from the perspective of accurately measuring the actual use on farms, the analysis was based on sales data, which does not directly reflect the amount of antimicrobial used in the animals. Since the sales data do not account for factors such as on-farm stock or disposal, the actual use on farms may differ from the sales figures.

In broilers, the decreasing trend, which could not be confirmed by sales amount alone, was detected using mg/kg biomass. Thus, mg/kg biomass is considered to be suitable for observing the changes in AMU in the animals over time, rather than for comparing this between animals. To that end, it is important to continue monitoring mg/kg biomass for each animal. There was an increasing trend in mg/kg biomass in beef cattle and dairy cattle and a decreasing trend in broilers, layers, and pigs (from 2017). According to Table 1, the oral administration rates for antimicrobial agents were 80% for beef cattle and 70% for dairy cattle. Conversely, broilers, layers, and pigs had oral administration rates above 95%. This may be because beef and dairy cattle are treated individually, such as injection and udder infusion in conjunction with herd treatment. In contrast, herd treatment seems to be a primary treatment method in other animal species; decreasing trends were observed in livestock species primarily treated in groups. This may be the result of veterinarians and farmers reassessing unnecessary oral administration in herds due to the recent campaigns regarding the prudent use of antibiotics. As biomass estimation methods in this study vary among animal species and cannot be accurately compared between animals using mg/kg biomass, it could still be estimated that AMU in fish raised in seawater and swine are higher than in other livestock. Though it was reported that the antimicrobial agent use in pigs was comparatively high [26], this study indicates that antimicrobial agent use is also high in marine aquaculture, in terms of both the mg/kg biomass index, as well as the sales amount of antimicrobials. As for the biomass of fish raised in seawater, there are variations in the types of fish farmed, and in some fish species farmed for several years, such as yellow tail and bluefin fish, the annual production amount may be underestimated. However, we believe that the method used in this study is the best effort to assess the biomass of fish raised in seawater using the most reliable and available statistics at present in Japan. In addition, fish are raised in large numbers within fish pens, making individual treatment impossible, meaning that if any abnormalities are observed within the group, it is often necessary to treat the entire group at once.

## 4. Materials and Methods

### 4.1. The Sales Amounts of Antimicrobial Agents for Therapeutic Use

The data were obtained from annual reports of sales amounts and volumes of antibiotics, synthetic antibacterials, anthelmintics, and antiprotozoals [27] in JVARM. Sales volumes are reported from marketing authorization holders in accordance with the Pharmaceutical and Medical Device Act. The classification of antimicrobials used in this study is described in Table 3.

### 4.2. Biomass Calculations

To calculate the biomass, we obtained the data mainly from the statistics published by the Ministry of Agriculture, Forestry and Fisheries of Japan (MAFF). Exceptionally, the average weight of the dairy cattle was obtained from the statistics published by the National Dairy Herd Improvement Association of Japan [19]. The basic concept of biomass in this study is product weight. However, as the production weights of dairy cattle and layers are not directly available, the biomasses of these animals were calculated by combining two statistics (the number of heads multiplied by the average weight). The breakdown of the data source and the methods of calculation are described in Table 4. The biomasses of the beef cattle and pigs were determined by the carcass weight, being the whole-body weight of a slaughtered animal after bleeding, evisceration, and skinning. Since the numbers of heads of layers in 2015 and 2020 were not available due to the census schedule, we interpolated the values, assuming that the number in 2015 was the same as in the previous year (2014) and assumed the same for the 2020 data.

### 4.3. Statistical Analyses for Trends

We performed the Mann–Kendall trend test to assess the presence of a trend in the data and computed Sen’s slope for the linear rate of change using a package named “trend” (Version 1.1.6) [32] with the R programming language (Version 4.3.0). For the purpose of this study, a probability of *p* < 0.05 was considered statistically significant. Beginning with the preprocessing steps, data were standardized to eliminate the difference in units and make the results comparable between the sales data and mg/kg biomass data. To standardize the data, we transformed each variable to have a mean of 0 and a standard deviation of 1. Data wrangling was performed using the “pandas” package (Version 1.5.3) [33] in Python (Version 3.10.6).

### 4.4. Forecasting

We used a package named “forecast” (Version 8.22.0) [34] to employ an automatic ARIMA model, an exponential smoothing state space model, and a linear model to forecast the sales amounts of the antimicrobial agents.

## 5. Conclusions

In this study, the status of AMU in Japan was assessed, and analyses were performed for each species by calculating its own biomass from reliable statistics within a selected timeframe. We believe that this indicator is stable, transparent, and easily understandable for national monitoring. By calculating the mg/kg biomass, it became possible to create an interpretation that is different from that of the simple sales quantity data. This allowed for the development of an index that considers population changes in the livestock industry and the occurrence of the disease in the designated timeline. There were increasing trends in mg/kg biomass in beef and dairy cattle, as well as in fish raised in seawater, while there were decreasing trends in broilers, layers, and fish raised in freshwater. In pigs, a decreasing trend was shown from 2017. In the broilers, it was clarified that AMU has decreased by considering the biomass despite the sales values remaining consistently stable. Antimicrobials are an important means of treating bacterial infections and must be administered carefully to ensure that currently used antimicrobial agents remain effective and can be used for treatment.

## Figures and Tables

**Table 1:** Quantities of veterinary antimicrobial agents sold for therapeutic use in Japan in tons of active ingredients by animals, with the proportions of oral administration.

Animal		2011	2012	2013	2014	2015	2016	2017	2018	2019	2020	2021	2022
Beef cattle	Tons	29.0	22.2	23.0	20.3	23.8	25.0	25.9	33.2	33.4	58.3	59.3	58.0
	%Oral	71.8	63.8	64.9	64.6	57.9	58.1	57.6	62.1	62.7	80.8	80.0	79.8
Dairy cattle	Tons	37.2	30.2	31.7	30.5	32.5	35.1	34.5	41.0	36.8	48.7	48.0	45.3
	%Oral	63.6	59.2	60.1	59.3	57.2	56.3	59.0	63.2	60.7	71.0	70.0	69.2
Pig	Tons	490.7	489.5	502.6	490.4	503.1	513.9	541.6	471.4	450.2	421.3	410.5	391.6
	%Oral	95.9	95.8	96.2	96.0	96.4	96.3	96.6	96.4	96.7	96.7	96.5	96.6
Broiler	Tons	62.9	73.4	65.9	70.1	62.4	63.8	61.7	62.8	69.8	77.5	69.1	61.5
	%Oral	99.1	98.4	98.7	98.8	98.6	98.6	98.5	98.5	98.8	98.9	98.8	98.7
Layer	Tons	32.8	21.9	23.3	23.7	19.4	19.8	15.3	15.9	17.6	17.1	9.3	9.7
	%Oral	99.5	98.8	99.1	99.2	99.2	99.3	99.1	99.2	99.3	99.2	98.4	98.8
Fish raised in freshwater	Tons	12.9	13.2	6.8	5.6	7.3	10.1	9.1	2.9	2.7	2.3	2.0	2.2
	%Oral	100.0	100.0	99.8	99.6	99.9	99.8	99.9	99.6	99.6	99.5	98.7	99.6
Fish raised in seawater	Tons	117.1	113.9	112.4	93.4	123.0	143.0	159.1	164.0	217.7	204.1	190.6	197.3
	%Oral	100.0	98.4	97.9	99.0	100.0	100.0	100.0	100.0	100.0	100.0	100.0	100.0
Total	Tons	782.6	764.3	765.8	734.0	771.4	810.7	847.3	791.1	828.2	829.4	788.8	765.5
	%Oral	94.5	94.2	94.4	94.4	94.4	94.4	94.8	94.2	94.8	95.1	94.7	94.8

**Table 2:** Biomasses of livestock animals in Japan in 1000 tons.

Animal	2011	2012	2013	2014	2015	2016	2017	2018	2019	2020	2021	2022
Beef cattle	500.4	518.6	508.0	502.1	481.0	464.4	469.1	475.3	470.8	477.5	477.6	491.3
Dairy cattle	930.1	917.2	902.2	883.0	870.6	854.1	842.8	848.6	851.1	863.9	865.1	876.1
Pig	1267.3	1297	1309.4	1263.6	1254.3	1278.6	1272.3	1284.1	1278.8	1306.0	1318.2	1293.4
Broiler	1783.4	1889.2	1905.3	1946.4	1984.0	2020.0	2063.2	2094.3	2143.1	2173.6	2225.6	2224.1
Layer	309.4	312.9	298.1	310.6	310.6	310.4	315.5	322.1	323.2	319.6	319.9	315.9
Fish raised in freshwater	38.9	34.0	30.5	33.9	36.3	35.2	36.8	29.8	31.2	29.1	32.9	31.4
Fish raised in seawater	231.6	250.5	243.7	238.0	246.1	247.6	247.6	249.5	248.1	251.9	256.2	236.6
Total	5061.0	5219.3	5197.1	5177.6	5183.0	5210.2	5247.3	5303.8	5346.4	5421.5	5495.4	5468.8

**Table 3:** The classifications of antimicrobials in this study.

Antimicrobial Class	Antimicrobials
Tetracyclines	Chlortetracycline, Doxycycline, Oxytetracycline
Macrolides	Erythromycin, Gamithromycin, Josamycin, Mirosamycin, Terdecamycin, Tildipirosin, Tilmicosin, Tulathromycin, Tylosin, Tylvalosin
Penicillins	Amoxicillin, Ampicillin, Aspoxicillin, Benzylpenicillin, Cloxacillin, Dicloxacillin, Mecillinam, Nafcillin, Tobicillin
Sulfonamides	Sulfachlorpyridazine, Sulfadimethoxine, Sulfadimidine, Sulfadoxine, Sulfamethoxazole, Sulfamonomethoxine, Sulfamoyldapsone, Sulfaquinoxaline, Sulfisozole
Aminoglycosides	Apramycin, Dihydrostreptomycin, Fradiomycin (Neomycin), Gentamicin, Kanamycin, Spectinomycin, Streptomycin
Thiamphenicols	Florfenicol, Thiamphenicol
Lincosamides	Lincomycin, Pirlimycin
Peptides	Colistin
Other synthetic antibacterials	Miloxacin, Ormetoprim, Trimethoprim
Fluoroquinolones	Danofloxacin, Difloxacin, Enrofloxacin, Marbofloxacin, Norfloxacin, Ofloxacin, Orbifloxacin
Cephalosporins	Cefalonium, Cefapirin, Cefazolin, Cefquinome, Ceftiofur, Cefuroxime
Other quinolones	Oxolinic acid
Antifungals	Nanafrocin
Furans	Nifurstyrenic acid
Others	Bicozamycin, Fosfomycin, Tiamulin, Valnemulin

**Table 4:** Data sources on biomass.

Animal Species	Data Source	Calculation
Beef cattle	Carcass weight from the Farm products distribution statistics, MAFF [28]	Carcass weight
Dairy cattle	1. Number of heads, Livestock statistics, MAFF [29] 2. Average live weight from the Dairy Herd Performance Records by the National Dairy Herd Improvement Association of Japan [30]	Number of heads(1) × Average live weight(2)
Pig	Carcass weight from the Farm products distribution statistics, MAFF [28]	Carcass weight
Broiler	Live weights at time of slaughter from the Farm products distribution statistics, MAFF [28]	Live weights at time of slaughter
Layer	1. Number of heads from the Livestock statistics, MAFF [29] 2. Number of heads for slaughter and live weights at the time of slaughter from the Farm products distribution statistics, MAFF [28]	$Average\;live\;weight\;=\;Live\;weights\;at\;time\;of\;slaughter\left(2\right)\;÷\;Number\;of\;heads\;for\;slaughter(2)$ $Number\;of\;heads\left(1\right)\;\times \;Average\;live\;weight$
Fish raised in seawater	Production volume from the Statistical Survey on Marine Fishery Production, MAFF [31]	Production volume
Fish raised in freshwater	Production volume from the Statistical Survey on Marine Fishery Production, MAFF [31]	Production volume

## Data Availability

The data that support the findings of this study are detailed in the Materials and Methods section of this article. Specific data sources and access information are provided within the references section.

## Supplementary Materials

The following supporting information can be downloaded at: https://www.mdpi.com/article/10.3390/antibiotics13121204/s1, Supplemental Table S1. Sales volume of antimicrobials by class and animals in kg. Supplemental Table S2. The weight of the active ingredient of veterinary antimicrobials divided by biomass per animal species (mg/kg biomass) for each antimicrobial class.
