# Supplementary material for: Antimicrobial Use in the Animal Sector in Japan from 2011 to 2022
Source: Antibiotics (Basel). 2024 Dec 10;13(12):1204. doi: 10.3390/antibiotics13121204 (PMC11672569; doi:10.3390/antibiotics13121204)
Supplement: Supplementary file 1 [file antibiotics-13-01204-s001.zip › antibiotics-3325416-supplementary.pdf]

Supplemental Table S1. Sales volume of Antimicrobials by class and animals in kg.

Antimicrobial class	Animal	2011	2012	2013	2014	2015	2016	2017	2018	2019	2020	2021	2022
Tetracyclines	Beef cattle	10632.9	8786.0	8478.6	6248.7	6236.9	6334.2	6719.3	12036.1	12365.0	33689.8	35690.6	35112.2
Tetracyclines	Dairy cattle	14004.9	13104.4	12966.2	11508.3	11532.2	12038.3	12481.4	18522.2	14600.0	23216.3	24045.0	22790.0
Tetracyclines	Pig	246523.4	244323.8	237081.8	232902.5	236277.3	239121.4	244313.2	198543.2	181906.4	152316.7	147256.6	135435.4
Tetracyclines	Broiler	23661.3	25768.4	24374.4	19445.1	17236.9	17815.2	18836.2	22651.7	27487.1	25706.6	26191.4	22574.8
Tetracyclines	Layer	6693.1	3975.9	3816.3	5726.7	4955.7	5354.5	3659.9	5606.0	6570.3	5180.1	3293.2	4772.8
Tetracyclines	Fish raised in freshwater	58.6	48.7	435.5	40.2	40.8	2.0	0	0	0	198.6	212.6	212.2
Tetracyclines	Fish raised in seawater	65593.4	59476.4	53348.1	48974.0	57578.7	50884.2	61045.0	52547.0	69572.5	63630.1	68609.4	79055.3
Tetracyclines	Total	367167.6	355483.6	340501.0	324845.4	333858.4	331549.7	347055.0	309906.2	312501.3	303938.2	305298.8	299952.7
Macrolides	Beef cattle	924.5	711.8	715.6	708.5	962.4	1082.1	1194.2	2467.9	2479.8	2709.3	2697.2	2547.4
Macrolides	Dairy cattle	736.4	492.3	517.4	545.5	792.6	894.1	810.0	1440.1	1364.5	1618.9	1577.8	1430.7
Macrolides	Pig	34328.9	36076.9	38054.2	36791.5	47943.9	58568.5	58985.0	57732.0	56898.4	57147.8	58695.4	50478.7
Macrolides	Broiler	11366.4	11304.3	10097.8	9000.1	7757.0	8975.5	8670.6	8251.2	10305.8	9239.1	8464.1	5713.0
Macrolides	Layer	6332.8	6518.0	6608.0	6245.0	2900.6	3154.7	2299.0	2197.8	2246.1	1998.3	1594.9	832.9
Macrolides	Fish raised in freshwater	0	0	167.2	0	0	0	0	0	0	0	0	0
Macrolides	Fish raised in seawater	22666.0	21370.8	21532.2	17130.4	38046.0	61436.0	68870.0	82610.0	107400.0	101010.0	84692.0	73684.0
Macrolides	Total	76355.0	76474.1	77692.3	70421.1	98402.6	134110.9	140828.8	154698.9	180694.6	173723.4	157721.4	134686.7
Penicillins	Beef cattle	7982.2	5361.1	5892.5	6167.8	7149.0	8002.1	7582.3	7318.1	7235.7	7156.1	7441.1	7143.0
Penicillins	Dairy cattle	9612.3	6710.9	7340.6	7495.7	8412.8	9325.8	9115.5	8291.6	8089.0	7426.7	7555.2	7342.5
Penicillins	Pig	37723.1	30300.1	30095.7	30704.5	33277.1	35748.3	35118.3	39761.7	38299.4	42442.7	40947.7	41093.0
Penicillins	Broiler	11672.0	9876.9	10086.5	10564.5	11099.2	12156.8	12734.9	11598.8	11973.3	11683.8	14209.8	14905.0
Penicillins	Layer	9961.1	4476.0	5079.6	6158.9	6643.5	7813.5	6504.0	5529.0	6058.5	5698.1	1484.1	1679.2
Penicillins	Fish raised in freshwater	0	0	64.9	0	0	0	495.0	0	0	0	0	0
Penicillins	Fish raised in seawater	15948.0	14655.7	16247.5	13869.3	14381.5	14618.0	14164.5	12853.0	17008.0	19214.0	14287.0	16162.0
Penicillins	Total	92898.7	71380.6	74807.3	74960.8	80963.0	87664.5	85714.5	85352.0	88663.8	93621.5	85925.0	88324.7
Sulfonamides	Beef cattle	5723.3	3416.4	4062.1	3942.1	3979.4	4274.8	4268.7	4347.7	4190.5	7486.4	5681.6	5852.8
Sulfonamides	Dairy cattle	5906.9	3789.9	4642.0	4394.5	4393.5	4849.3	4827.9	5025.4	4869.3	7654.4	5648.8	5201.5
Sulfonamides	Pig	66204.5	70512.2	73142.4	60877.5	59152.7	57399.1	65260.7	60778.4	52059.6	51688.6	47166.8	45975.4
Sulfonamides	Broiler	4727.4	6202.7	6123.9	13427.5	11664.0	7943.6	6302.5	5399.0	4563.8	12540.6	3085.3	3075.3
Sulfonamides	Layer	4468.0	5752.1	6288.3	4499.2	3903.3	2749.8	2242.8	1907.1	1957.5	3339.6	1946.0	1602.9
Sulfonamides	Fish raised in freshwater	12694.3	12939.9	5894.4	5237.1	6756.1	9675.6	7995.3	2642.6	2165.1	1639.1	1485.7	1640.6
Sulfonamides	Fish raised in seawater	3149.8	3194.0	1455.6	2965.5	4644.1	6753.9	6104.6	6808.1	13200.4	11479.9	15712.5	19504.4
Sulfonamides	Total	102874.2	105807.2	101608.6	95343.3	94493.3	93646.0	97002.5	86908.4	83006.1	95828.6	80726.7	82852.9
Aminoglycosides	Beef cattle	1131.6	1044.8	1059.9	1014.7	1773.2	1759.2	1681.1	1875.7	1738.4	1539.5	1495.3	1392.4
Aminoglycosides	Dairy cattle	3301.1	2254.3	2307.0	2251.8	3073.9	2916.4	2758.1	2968.5	2872.9	2981.8	2940.0	2843.8
Aminoglycosides	Pig	18190.9	23679.9	23637.6	24462.2	20773.7	30583.2	29562.0	20449.0	21898.8	21318.8	16197.3	17701.0
Aminoglycosides	Broiler	4751.4	11609.8	9725.2	10224.6	8131.7	11810.0	9970.5	8507.6	7761.4	10214.3	8606.6	8835.7
Aminoglycosides	Layer	4034.4	235.0	163.9	139.5	122.0	118.9	115.8	105.3	108.3	118.7	125.6	120.0
Aminoglycosides	Fish raised in freshwater	0	0	0	0	0	0	0	0	0	0	0	0
Aminoglycosides	Fish raised in seawater	0	0	0	0	0	0	0	0	0	0	0	0
Aminoglycosides	Total	31409.5	38823.9	36893.7	38092.8	33874.5	47187.7	44087.4	33906.2	34379.9	36173.0	29364.8	30892.9
Thiamphenicols	Beef cattle	1597.9	1791.1	1605.4	953.4	2187.5	1785.4	2592.1	3107.7	3260.1	3038.1	3163.2	3498.1
Thiamphenicols	Dairy cattle	556.7	637.0	689.2	973.4	785.3	1480.5	1015.6	915.3	1009.5	1059.3	949.9	1032.6
Thiamphenicols	Pig	16000.6	15391.2	14713.3	19050.2	22850.8	20735.9	20708.1	16933.3	17829.3	17370.5	18651.4	19574.0
Thiamphenicols	Broiler	680.6	689.5	890.2	1676.5	1571.0	814.6	1021.6	2209.2	1691.7	1529.4	1370.9	1077.1
Thiamphenicols	Layer	0	0	0	0	0	0	0	114.5	103.4	116.0	89.8	89.5
Thiamphenicols	Fish raised in freshwater	0	20.0	34.6	70.4	159.3	155.5	413.4	157.3	241.4	197.9	242.2	218.8
Thiamphenicols	Fish raised in seawater	2257.7	1432.3	1837.6	941.9	2171.2	1516.5	1355.3	1375.2	3235.9	2230.5	2541.8	2775.2
Thiamphenicols	Total	21093.5	19961.1	19770.3	23665.8	29725.1	26488.4	27106.0	24812.6	27371.3	25541.6	27009.1	28265.2
Lincosamides	Beef cattle	0	0	0	0	0	0	0	0	0	113.4	128.0	141.9
Lincosamides	Dairy cattle	0	0	0	0	0	4.5	5.2	5.8	6.2	376.5	408.4	423.1
Lincosamides	Pig	32822.7	33443.8	34427.6	35410.7	23106.8	15059.3	18790.0	16129.4	15730.7	16319.8	17834.9	18409.0

Lincosamides	Broiler	1987.0	5022.6	1452.7	1200.3	540.5	555.2	599.0	580.8	519.7	669.5	731.4	622.1
Lincosamides	Layer	0	0	0	0	0	0	0	0	0	2.5	3.1	3.2
Lincosamides	Fish raised in freshwater	0	0	0	0	0	0	0	0	0	0	0	0
Lincosamides	Fish raised in seawater	3810.0	4222.4	3024.0	6558.6	4902.0	6120.0	5728.0	5908.0	4882.0	3816.0	3190.0	3940.0
Lincosamides	Total	38619.7	42688.7	38904.3	43169.5	28549.4	21739.0	25122.2	22624.0	21138.6	21297.7	22295.8	23539.3
Peptides	Beef cattle	0	0	0	0	0	0	0	0	0	0	0	0
Peptides	Dairy cattle	0	0	0	0	0	0	0	0.7	0.2	0	0	0
Peptides	Pig	5688.2	8537.9	11769.4	9971.1	14537.5	14012.0	19980.2	11831.3	18944.1	18410.0	17744.8	17428.1
Peptides	Broiler	0	0	0	0	0	0	0	503.0	613.6	639.0	648.5	1113.7
Peptides	Layer	0	0	0	0	0	0	0	0	0	0	0	0
Peptides	Fish raised in freshwater	0	0	0	0	0	0	0	0	0	0	0	0
Peptides	Fish raised in seawater	0	0	0	0	0	0	0	0	0	0	0	0
Peptides	Total	5688.2	8537.9	11769.4	9971.1	14537.5	14012.0	19980.2	12335.0	19557.9	19049.0	18393.4	18541.8
Other synthetic antibacterials	Beef cattle	403.1	373.0	389.6	410.4	436.0	492.2	477.8	489.5	523.5	959.1	1229.9	628.0
Other synthetic antibacterials	Dairy cattle	403.1	373.0	389.6	410.4	436.0	492.2	477.8	491.9	526.0	829.4	1115.0	514.3
Other synthetic antibacterials	Pig	11251.3	11805.3	12316.9	10076.7	9864.3	9486.2	10914.2	10037.2	8479.8	7334.4	6633.1	7871.7
Other synthetic antibacterials	Broiler	671.6	717.1	898.9	2407.1	2072.3	1314.1	998.0	827.5	2003.9	2150.9	2109.1	439.8
Other synthetic antibacterials	Layer	803.7	681.6	982.5	618.2	510.1	285.2	150.3	109.8	147.4	257.5	324.7	245.1
Other synthetic antibacterials	Fish raised in freshwater	31.7	42.9	24.0	36.8	16.2	37.8	58.3	17.7	22.8	30.7	42.2	62.6
Other synthetic antibacterials	Fish raised in seawater	0	0	0	0	0	0	0	0	0	93.8	91.7	93.0
Other synthetic antibacterials	Total	13564.5	13992.9	15001.5	13959.5	13334.9	12107.7	13076.3	11973.5	11703.5	11655.7	11545.7	9854.5
Fluoroquinolones	Beef cattle	276.3	348.8	359.8	462.4	549.6	735.8	890.9	957.3	1058.1	1041.0	1053.3	1068.4
Fluoroquinolones	Dairy cattle	413.0	421.2	346.8	466.6	522.8	533.3	426.2	419.7	433.9	520.1	540.1	609.7
Fluoroquinolones	Pig	1988.9	1409.3	1431.6	1529.8	2826.6	1232.5	1918.8	1877.8	1938.9	1118.3	1915.9	1772.0
Fluoroquinolones	Broiler	3237.5	2107.3	2169.1	1991.9	2211.3	2392.0	2346.3	2258.4	2866.7	3077.6	3569.6	2921.2
Fluoroquinolones	Layer	491.3	301.7	329.3	279.6	299.5	300.3	343.6	289.5	364.0	423.0	462.9	331.6
Fluoroquinolones	Fish raised in freshwater	0	0	0	0	0	0	0	0	0	0	0	0
Fluoroquinolones	Fish raised in seawater	0	0	0	0	0	0	0	0	0	0	0	0
Fluoroquinolones	Total	6407.0	4588.3	4636.6	4730.4	6409.8	5193.8	5925.8	5802.8	6661.7	6179.9	7541.9	6702.8
Cephalosporins	Beef cattle	245.5	285.2	292.0	305.9	324.6	350.8	365.3	443.8	424.3	301.6	346.8	288.8
Cephalosporins	Dairy cattle	2230.2	2353.2	2471.5	2381.6	2470.3	2495.5	2583.6	2861.7	2941.0	2837.7	3001.6	2972.0
Cephalosporins	Pig	374.4	360.6	358.2	372.7	425.8	489.2	487.5	601.2	742.5	647.7	704.7	724.4
Cephalosporins	Broiler	0	0	0	0	0	0	0	0	0	0	0	0
Cephalosporins	Layer	0	0	0	0	0	0	0	0	0	0	0	0
Cephalosporins	Fish raised in freshwater	0	0	0	0	0	0	0	0	0	0	0	0
Cephalosporins	Fish raised in seawater	0	0	0	0	0	0	0	0	0	0	0	0
Cephalosporins	Total	2850.1	2999.1	3121.7	3060.2	3220.7	3335.5	3436.4	3906.7	4107.8	3786.9	4053.1	3985.3
Other quinolones	Beef cattle	12.8	0	21.2	0.0100	16.0	17.3	7.4	1.9	12.0	14.3	2.8	1.9
Other quinolones	Dairy cattle	3.2	0	37.4	0.0240	25.1	28.2	13.2	5.7	21.0	24.0	8.3	5.7
Other quinolones	Pig	9.1	0	60.8	0.0020	59.3	75.5	26.0	1.0	51.0	63.4	1.4	1.0
Other quinolones	Broiler	111.8	98.7	80.5	198.9	79.7	37.7	261.3	1.0	26.0	81.6	150.5	249.0
Other quinolones	Layer	0	0	23.6	0.0020	22.4	0	0	0	0	0	0	0
Other quinolones	Fish raised in freshwater	122.6	126.1	216.1	218.1	312.3	227.5	111.4	94.8	314.0	201.6	44.1	19.5
Other quinolones	Fish raised in seawater	909.2	1187.5	528.9	1433.6	1141.0	1285.4	1333.4	1268.9	2044.6	1874.5	1275.0	1737.5
Other quinolones	Total	1168.8	1412.4	968.3	1850.6	1655.7	1671.6	1752.6	1373.2	2468.5	2259.3	1482.1	2014.5
Antifungals	Beef cattle	1.0	0.1	0.1	0.0810	0.2	0.2	0.3	0.3	0.3	0.3	0.3	0.3
Antifungals	Dairy cattle	1.0	0.1	0.1	0.2	0.0964	0.0990	0.0731	0.0740	0.0744	0.0694	0.0778	0.0736
Antifungals	Pig	0	0	0	0	0	0	0	0	0	0	0	0
Antifungals	Broiler	0	0	0	0	0	0	0	0	0	0	0	0

Antifungals	Layer	0	0	0	0	0	0	0	0	0	0	0	0
Antifungals	Fish raised in freshwater	0	0	0	0	0	0	0	0	0	0	0	0
Antifungals	Fish raised in seawater	0	0	0	0	0	0	0	0	0	0	0	0
Antifungals	Total	2.0	0.3	0.3	0.3	0.3	0.3	0.4	0.4	0.4	0.3	0.4	0.4
Furans	Beef cattle	0	0	0	0	0	0	0	0	0	0	0	0
Furans	Dairy cattle	0	0	0	0	0	0	0	0	0	0	0	0
Furans	Pig	0	0	0	0	0	0	0	0	0	0	0	0
Furans	Broiler	0	0	0	0	0	0	0	0	0	0	0	0
Furans	Layer	0	0	0	0	0	0	0	0	0	0	0	0
Furans	Fish raised in freshwater	0	0	0	0	0	0	0	0	0	0	0	0
Furans	Fish raised in seawater	2794.2	8179.0	14113.9	1128.5	0	0	0	0	0	0	0	0
Furans	Total	2794.2	8179.0	14113.9	1128.5	0	0	0	0	0	0	0	0
Others	Beef cattle	60.5	54.9	144.3	135.6	157.7	161.1	141.8	121.4	111.6	283.9	343.4	325.4
Others	Dairy cattle	42.5	38.5	18.3	26.3	37.1	39.4	31.0	64.9	61.1	167.4	183.2	178.5
Others	Pig	19618.7	13695.9	25550.1	28270.6	32032.0	31345.4	35545.5	36683.3	35463.5	35093.6	36768.8	35104.6
Others	Broiler	0	0	0	0	0	0	0	0	0	0	0	0
Others	Layer	0	0	0	0	0	0	0	0	0	0	0	0
Others	Fish raised in freshwater	0	0	0.2	3.2	0	0	0	0	0	0	0	0
Others	Fish raised in seawater	0	208.0	270.0	412.6	159.0	419.0	468.0	629.0	319.0	797.0	158.0	312.0
Others	Total	19721.7	13997.4	25983.0	28848.2	32385.8	31965.0	36186.3	37498.7	35955.1	36341.9	37453.5	35920.5
Total	Total	782614.8	764326.4	765772.3	734047.5	771410.8	810672.1	847274.2	791098.6	828210.3	829397.1	788811.5	765534.2

Supplemental Table S2. The weight of active ingredient of veterinary antimicrobials divided by biomass per animal species (mg/kg biomass) for each antimicrobial class.

Animal	Antimicrobial class	2011	2012	2013	2014	2015	2016	2017	2018	2019	2020	2021	2022
Beef cattle	Tetracyclines	21.3	16.9	16.7	12.4	13.0	13.6	14.3	25.3	26.3	70.6	74.7	71.5
Beef cattle	Macrolides	1.8	1.4	1.4	1.4	2.0	2.3	2.5	5.2	5.3	5.7	5.6	5.2
Beef cattle	Penicillins	16.0	10.3	11.6	12.3	14.9	17.2	16.2	15.4	15.4	15.0	15.6	14.5
Beef cattle	Sulfonamides	11.4	6.6	8.0	7.9	8.3	9.2	9.1	9.1	8.9	15.7	11.9	11.9
Beef cattle	Aminoglycosides	2.3	2.0	2.1	2.0	3.7	3.8	3.6	3.9	3.7	3.2	3.1	2.8
Beef cattle	Thiamphenicols	3.2	3.5	3.2	1.9	4.5	3.8	5.5	6.5	6.9	6.4	6.6	7.1
Beef cattle	Lincosamides	0.0	0.0	0.0	0.0	0.0	0.0	0.0	0.0	0.0	0.2	0.3	0.3
Beef cattle	Peptides	0.0	0.0	0.0	0.0	0.0	0.0	0.0	0.0	0.0	0.0	0.0	0.0
Beef cattle	Other synthetic antibacterials	0.8	0.7	0.8	0.8	0.9	1.1	1.0	1.0	1.1	2.0	2.6	1.3
Beef cattle	Fluoroquinolones	0.6	0.7	0.7	0.9	1.1	1.6	1.9	2.0	2.2	2.2	2.2	2.2
Beef cattle	Cephalosporins	0.5	0.5	0.6	0.6	0.7	0.8	0.8	0.9	0.9	0.6	0.7	0.6
Beef cattle	Other quinolones	0.0	0.0	0.0	0.0	0.0	0.0	0.0	0.0	0.0	0.0	0.0	0.0
Beef cattle	Antifungals	0.0	0.0	0.0	0.0	0.0	0.0	0.0	0.0	0.0	0.0	0.0	0.0
Beef cattle	Furans	0.0	0.0	0.0	0.0	0.0	0.0	0.0	0.0	0.0	0.0	0.0	0.0
Beef cattle	Others	0.1	0.1	0.3	0.3	0.3	0.3	0.3	0.3	0.2	0.6	0.7	0.7
Beef cattle	Total	57.9	42.8	45.3	40.5	49.4	53.8	55.3	69.8	70.9	122.2	124.1	118.1
Dairy cattle	Tetracyclines	15.1	14.3	14.4	13.0	13.2	14.1	14.8	21.8	17.2	26.9	27.8	26.0
Dairy cattle	Macrolides	0.8	0.5	0.6	0.6	0.9	1.0	1.0	1.7	1.6	1.9	1.8	1.6
Dairy cattle	Penicillins	10.3	7.3	8.1	8.5	9.7	10.9	10.8	9.8	9.5	8.6	8.7	8.4
Dairy cattle	Sulfonamides	6.4	4.1	5.1	5.0	5.0	5.7	5.7	5.9	5.7	8.9	6.5	5.9
Dairy cattle	Aminoglycosides	3.5	2.5	2.6	2.6	3.5	3.4	3.3	3.5	3.4	3.5	3.4	3.2
Dairy cattle	Thiamphenicols	0.6	0.7	0.8	1.1	0.9	1.7	1.2	1.1	1.2	1.2	1.1	1.2
Dairy cattle	Lincosamides	0.0	0.0	0.0	0.0	0.0	0.0	0.0	0.0	0.0	0.4	0.5	0.5
Dairy cattle	Peptides	0.0	0.0	0.0	0.0	0.0	0.0	0.0	0.0	0.0	0.0	0.0	0.0
Dairy cattle	Other synthetic antibacterials	0.4	0.4	0.4	0.5	0.5	0.6	0.6	0.6	0.6	1.0	1.3	0.6
Dairy cattle	Fluoroquinolones	0.4	0.5	0.4	0.5	0.6	0.6	0.5	0.5	0.5	0.6	0.6	0.7

Dairy cattle	Cephalosporins	2.4	2.6	2.7	2.7	2.8	2.9	3.1	3.4	3.5	3.3	3.5	3.4
Dairy cattle	Other quinolones	0.0	0.0	0.0	0.0	0.0	0.0	0.0	0.0	0.0	0.0	0.0	0.0
Dairy cattle	Antifungals	0.0	0.0	0.0	0.0	0.0	0.0	0.0	0.0	0.0	0.0	0.0	0.0
Dairy cattle	Furans	0.0	0.0	0.0	0.0	0.0	0.0	0.0	0.0	0.0	0.0	0.0	0.0
Dairy cattle	Others	0.0	0.0	0.0	0.0	0.0	0.0	0.0	0.1	0.1	0.2	0.2	0.2
Dairy cattle	Total	40.0	32.9	35.2	34.5	37.3	41.1	41.0	48.3	43.2	56.4	55.5	51.8
Pig	Tetracyclines	194.5	188.4	181.1	184.3	188.4	187.0	192.0	154.6	142.2	116.6	111.7	104.7
Pig	Macrolides	27.1	27.8	29.1	29.1	38.2	45.8	46.4	45.0	44.5	43.8	44.5	39.0
Pig	Penicillins	29.8	23.4	23.0	24.3	26.5	28.0	27.6	31.0	29.9	32.5	31.1	31.8
Pig	Sulfonamides	52.2	54.4	55.9	48.2	47.2	44.9	51.3	47.3	40.7	39.6	35.8	35.5
Pig	Aminoglycosides	14.4	18.3	18.1	19.4	16.6	23.9	23.2	15.9	17.1	16.3	12.3	13.7
Pig	Thiamphenicols	12.6	11.9	11.2	15.1	18.2	16.2	16.3	13.2	13.9	13.3	14.1	15.1
Pig	Lincosamides	25.9	25.8	26.3	28.0	18.4	11.8	14.8	12.6	12.3	12.5	13.5	14.2
Pig	Peptides	4.5	6.6	9.0	7.9	11.6	11.0	15.7	9.2	14.8	14.1	13.5	13.5
Pig	Other synthetic antibacterials	8.9	9.1	9.4	8.0	7.9	7.4	8.6	7.8	6.6	5.6	5.0	6.1
Pig	Fluoroquinolones	1.6	1.1	1.1	1.2	2.3	1.0	1.5	1.5	1.5	0.9	1.5	1.4
Pig	Cephalosporins	0.3	0.3	0.3	0.3	0.3	0.4	0.4	0.5	0.6	0.5	0.5	0.6
Pig	Other quinolones	0.0	0.0	0.0	0.0	0.0	0.1	0.0	0.0	0.0	0.0	0.0	0.0
Pig	Antifungals	0.0	0.0	0.0	0.0	0.0	0.0	0.0	0.0	0.0	0.0	0.0	0.0
Pig	Furans	0.0	0.0	0.0	0.0	0.0	0.0	0.0	0.0	0.0	0.0	0.0	0.0
Pig	Others	15.5	10.6	19.5	22.4	25.5	24.5	27.9	28.6	27.7	26.9	27.9	27.1
Pig	Total	387.2	377.4	383.9	388.1	401.1	401.9	425.7	367.1	352.1	322.6	311.4	302.7
Broiler	Tetracyclines	13.3	13.6	12.8	10.0	8.7	8.8	9.1	10.8	12.8	11.8	11.8	10.1
Broiler	Macrolides	6.4	6.0	5.3	4.6	3.9	4.4	4.2	3.9	4.8	4.3	3.8	2.6
Broiler	Penicillins	6.5	5.2	5.3	5.4	5.6	6.0	6.2	5.5	5.6	5.4	6.4	6.7
Broiler	Sulfonamides	2.7	3.3	3.2	6.9	5.9	3.9	3.1	2.6	2.1	5.8	1.4	1.4
Broiler	Aminoglycosides	2.7	6.1	5.1	5.3	4.1	5.8	4.8	4.1	3.6	4.7	3.9	4.0
Broiler	Thiamphenicols	0.4	0.4	0.5	0.9	0.8	0.4	0.5	1.1	0.8	0.7	0.6	0.5
Broiler	Lincosamides	1.1	2.7	0.8	0.6	0.3	0.3	0.3	0.3	0.2	0.3	0.3	0.3
Broiler	Peptides	0.0	0.0	0.0	0.0	0.0	0.0	0.0	0.2	0.3	0.3	0.3	0.5
Broiler	Other synthetic antibacterials	0.4	0.4	0.5	1.2	1.0	0.7	0.5	0.4	0.9	1.0	0.9	0.2
Broiler	Fluoroquinolones	1.8	1.1	1.1	1.0	1.1	1.2	1.1	1.1	1.3	1.4	1.6	1.3
Broiler	Cephalosporins	0.0	0.0	0.0	0.0	0.0	0.0	0.0	0.0	0.0	0.0	0.0	0.0
Broiler	Other quinolones	0.1	0.1	0.0	0.1	0.0	0.0	0.1	0.0	0.0	0.0	0.1	0.1
Broiler	Antifungals	0.0	0.0	0.0	0.0	0.0	0.0	0.0	0.0	0.0	0.0	0.0	0.0
Broiler	Furans	0.0	0.0	0.0	0.0	0.0	0.0	0.0	0.0	0.0	0.0	0.0	0.0
Broiler	Others	0.0	0.0	0.0	0.0	0.0	0.0	0.0	0.0	0.0	0.0	0.0	0.0
Broiler	Total	35.3	38.9	34.6	36.0	31.4	31.6	29.9	30.0	32.6	35.7	31.1	27.7
Layer	Tetracyclines	21.6	12.7	12.8	18.4	16.0	17.2	11.6	17.4	20.3	16.2	10.3	15.1
Layer	Macrolides	20.5	20.8	22.2	20.1	9.3	10.2	7.3	6.8	6.9	6.3	5.0	2.6
Layer	Penicillins	32.2	14.3	17.0	19.8	21.4	25.2	20.6	17.2	18.7	17.8	4.6	5.3
Layer	Sulfonamides	14.4	18.4	21.1	14.5	12.6	8.9	7.1	5.9	6.1	10.5	6.1	5.1
Layer	Aminoglycosides	13.0	0.8	0.5	0.4	0.4	0.4	0.4	0.3	0.3	0.4	0.4	0.4
Layer	Thiamphenicols	0.0	0.0	0.0	0.0	0.0	0.0	0.0	0.4	0.3	0.4	0.3	0.3
Layer	Lincosamides	0.0	0.0	0.0	0.0	0.0	0.0	0.0	0.0	0.0	0.0	0.0	0.0
Layer	Peptides	0.0	0.0	0.0	0.0	0.0	0.0	0.0	0.0	0.0	0.0	0.0	0.0
Layer	Other synthetic antibacterials	2.6	2.2	3.3	2.0	1.6	0.9	0.5	0.3	0.5	0.8	1.0	0.8
Layer	Fluoroquinolones	1.6	1.0	1.1	0.9	1.0	1.0	1.1	0.9	1.1	1.3	1.4	1.0
Layer	Cephalosporins	0.0	0.0	0.0	0.0	0.0	0.0	0.0	0.0	0.0	0.0	0.0	0.0
Layer	Other quinolones	0.0	0.0	0.1	0.0	0.1	0.0	0.0	0.0	0.0	0.0	0.0	0.0
Layer	Antifungals	0.0	0.0	0.0	0.0	0.0	0.0	0.0	0.0	0.0	0.0	0.0	0.0

Layer	Furans	0.0	0.0	0.0	0.0	0.0	0.0	0.0	0.0	0.0	0.0	0.0	0.0
Layer	Others	0.0	0.0	0.0	0.0	0.0	0.0	0.0	0.0	0.0	0.0	0.0	0.0
Layer	Total	106.0	70.1	78.1	76.2	62.3	63.7	48.5	49.2	54.3	53.6	29.1	30.6
Fish raised in freshwater	Tetracyclines	1.5	1.4	14.3	1.2	1.1	0.1	0.0	0.0	0.0	6.8	6.5	6.8
Fish raised in freshwater	Macrolides	0.0	0.0	5.5	0.0	0.0	0.0	0.0	0.0	0.0	0.0	0.0	0.0
Fish raised in freshwater	Penicillins	0.0	0.0	2.1	0.0	0.0	0.0	13.4	0.0	0.0	0.0	0.0	0.0
Fish raised in freshwater	Sulfonamides	326.4	381.1	193.3	154.6	185.9	274.9	217.0	88.5	69.4	56.4	45.2	52.3
Fish raised in freshwater	Aminoglycosides	0.0	0.0	0.0	0.0	0.0	0.0	0.0	0.0	0.0	0.0	0.0	0.0
Fish raised in freshwater	Thiamphenicols	0.0	0.6	1.1	2.1	4.4	4.4	11.2	5.3	7.7	6.8	7.4	7.0
Fish raised in freshwater	Lincosamides	0.0	0.0	0.0	0.0	0.0	0.0	0.0	0.0	0.0	0.0	0.0	0.0
Fish raised in freshwater	Peptides	0.0	0.0	0.0	0.0	0.0	0.0	0.0	0.0	0.0	0.0	0.0	0.0
Fish raised in freshwater	Other synthetic antibacterials	0.8	1.3	0.8	1.1	0.4	1.1	1.6	0.6	0.7	1.1	1.3	2.0
Fish raised in freshwater	Fluoroquinolones	0.0	0.0	0.0	0.0	0.0	0.0	0.0	0.0	0.0	0.0	0.0	0.0
Fish raised in freshwater	Cephalosporins	0.0	0.0	0.0	0.0	0.0	0.0	0.0	0.0	0.0	0.0	0.0	0.0
Fish raised in freshwater	Other quinolones	3.2	3.7	7.1	6.4	8.6	6.5	3.0	3.2	10.1	6.9	1.3	0.6
Fish raised in freshwater	Antifungals	0.0	0.0	0.0	0.0	0.0	0.0	0.0	0.0	0.0	0.0	0.0	0.0
Fish raised in freshwater	Furans	0.0	0.0	0.0	0.0	0.0	0.0	0.0	0.0	0.0	0.0	0.0	0.0
Fish raised in freshwater	Others	0.0	0.0	0.0	0.1	0.0	0.0	0.0	0.0	0.0	0.0	0.0	0.0
Fish raised in freshwater	Total	331.9	388.1	224.2	165.5	200.5	286.9	246.3	97.6	87.9	78.0	61.7	68.6
Fish raised in seawater	Tetracyclines	283.2	237.5	218.9	205.8	234.0	205.5	246.5	210.6	280.4	252.6	267.8	334.1
Fish raised in seawater	Macrolides	97.9	85.3	88.4	72.0	154.6	248.1	278.1	331.1	432.8	401.0	330.6	311.4
Fish raised in seawater	Penicillins	68.9	58.5	66.7	58.3	58.4	59.0	57.2	51.5	68.5	76.3	55.8	68.3
Fish raised in seawater	Sulfonamides	13.6	12.8	6.0	12.5	18.9	27.3	24.7	27.3	53.2	45.6	61.3	82.4
Fish raised in seawater	Aminoglycosides	0.0	0.0	0.0	0.0	0.0	0.0	0.0	0.0	0.0	0.0	0.0	0.0
Fish raised in seawater	Thiamphenicols	9.7	5.7	7.5	4.0	8.8	6.1	5.5	5.5	13.0	8.9	9.9	11.7
Fish raised in seawater	Lincosamides	16.5	16.9	12.4	27.6	19.9	24.7	23.1	23.7	19.7	15.1	12.5	16.7
Fish raised in seawater	Peptides	0.0	0.0	0.0	0.0	0.0	0.0	0.0	0.0	0.0	0.0	0.0	0.0
Fish raised in seawater	Other synthetic antibacterials	0.0	0.0	0.0	0.0	0.0	0.0	0.0	0.0	0.0	0.4	0.4	0.4
Fish raised in seawater	Fluoroquinolones	0.0	0.0	0.0	0.0	0.0	0.0	0.0	0.0	0.0	0.0	0.0	0.0
Fish raised in seawater	Cephalosporins	0.0	0.0	0.0	0.0	0.0	0.0	0.0	0.0	0.0	0.0	0.0	0.0
Fish raised in seawater	Other quinolones	3.9	4.7	2.2	6.0	4.6	5.2	5.4	5.1	8.2	7.4	5.0	7.3
Fish raised in seawater	Antifungals	0.0	0.0	0.0	0.0	0.0	0.0	0.0	0.0	0.0	0.0	0.0	0.0
Fish raised in seawater	Furans	12.1	32.7	57.9	4.7	0.0	0.0	0.0	0.0	0.0	0.0	0.0	0.0
Fish raised in seawater	Others	0.0	0.8	1.1	1.7	0.6	1.7	1.9	2.5	1.3	3.2	0.6	1.3
Fish raised in seawater	Total	505.7	454.8	461.1	392.6	499.9	577.7	642.4	657.3	877.2	810.4	743.8	833.7
